# Effect of reduced formal care availability on formal/informal care patterns and caregiver health: a quasi-experimental study using the Japanese long-term care insurance reform

**DOI:** 10.1186/s12877-020-01588-7

**Published:** 2020-06-12

**Authors:** Atsushi Miyawaki, Yasuki Kobayashi, Haruko Noguchi, Taeko Watanabe, Hideto Takahashi, Nanako Tamiya

**Affiliations:** 1 Department of Public Health, Graduate School of Medicine, The University of Tokyo, 7-3-1 Hongo, Bunkyo-ku, Tokyo, 1130033 Japan; 2 Health Services Development and Research Center, University of Tsukuba, 1-1-1 Tennodai, Tsukuba-shi, Ibaraki, 3058577 Japan; 3 Faculty of Political Science and Economics, Waseda University, 1-6-1 Nishiwaseda Shinjuku-ku, Tokyo, 1698050 Japan; 4 Department of Health Services Research, Faculty of Medicine, University of Tsukuba, 1-1-1 Tennodai, Tsukuba-shi, Ibaraki, 3058577 Japan; 5 National Institute of Public Health, 2-3-6 Minami, Wako-shi, Saitama, 3510197 Japan

**Keywords:** Long-term care, Caregiving, Health care policy, Japan

## Abstract

**Background:**

It is unclear how formal long-term care (LTC) availability affects formal /informal caregiving patterns and caregiver health. We tested the impact of reduced formal LTC availability on formal LTC service use, intensity of informal caregiving, and caregiver health.

**Methods:**

Using a representative, repeated cross-sectional sample of Japanese caregivers providing care to co-resident family members from 2001 to 2016, we applied a difference-in-differences approach by observing caregivers before and after the major reform of the public Japanese LTC insurance (LTCI) in 2006. The reform reduced coverage benefits for non-institutionalized older persons with low care needs, but not for those with high care needs. We analyzed 12,764 caregivers aged ≥30 years (mean age 64.3 ± 11.8 years, 73.5% women) and measured indicators of formal LTC use, hours of informal caregiving, and caregiver self-reported health outcomes after propensity score matching to balance caregivers’ background characteristics.

**Results:**

We found the 2006 LTCI reform relatively reduced the use of formal LTC services and relatively increased the percentage of experiencing long hours of informal caregiving (> 3 h per day) among the caregivers for seniors with low care needs compared to those for seniors with high care needs. The effects of the LTCI reform for the caregivers for seniors with low care needs were 2.2 percentage point higher on caregivers’ experiencing poor self-rated health (95% confidence interval [CI]: 0.7–3.7; *p* = 0.01), 2.7 percentage point higher on experiencing symptoms of a depressive state (95%CI: 0.5–4.8; *p* = 0.03), and 4.7 percentage point higher on experiencing symptoms of musculoskeletal diseases (95%CI, 3.6–5.7; *p* < 0.001), compared to those for seniors with high care needs.

**Conclusions:**

Reduced formal care availability under the Japanese LTCI reform increased hours of informal caregiving corresponding to reduced use of formal LTC and deteriorated multiple dimensions of caregiver health. Our findings may highlight the importance of enhancing the availability of formal LTC services for caregiver health.

## Backgrounds

In the context of modern aging societies, several countries offer formal long-term care (LTC) services—provided by paid professionals—under universal public LTC programs. Nordic countries (Norway, Sweden, Denmark, and Finland), the UK, Ireland, Spain, and Australia have developed a tax-based model of formal LTC [1]. Japan, like Germany, the Netherlands, Korea, and Luxembourg, provides comprehensive formal LTC programs via a social insurance system known as long-term care insurance (LTCI) [2]. Formal LTC systems aim to “socialize” LTC burdens by taking over part of the responsibility of informal caregivers (unpaid people providing care to family members) for providing care to family members. This is assumed to mitigate the caregiving burden among informal caregivers and improve their well-being [3]. For example, “*10 priorities for a decade of action on healthy ageing*” is an initiative that was recently launched by the World Health Organization to encourage all countries to develop effective LTC systems to reduce caregiver burden [4].

However, it is still unknown whether formal LTC services availability improves caregiver health. Although several previous studies have demonstrated that formal LTC is a substitute for informal LTC [5–7], empirical studies have not reported obvious benefits of formal LTC use on caregiver health [8]. Moreover, the mechanism through which formal LTC availability affects informal caregiver health is not established because it remains inconclusive whether or not informal caregiving harms caregiver health. While a highly intensive level of caregiving may lead to an increased risk for depression [9], hypertension [10], and cardiovascular disease [11–13], informal caregiving may improve caregiver health via rewards or satisfaction from altruism [14–17]. Recent epidemiological studies from the UK and the US also report that informal caregiving is associated with decreased mortality [18, 19]. In summary, the net effect of formal LTC services availability on informal caregiver health is unclear empirically and structurally.

To address this knowledge gap, we sought to answer the following questions using a nationally representative sample of Japanese informal caregivers. First, does reduced formal LTC availability affect formal LTC service use and intensity of informal caregiving? Second, does reduced formal LTC availability have adverse effects on informal caregiver health?

## Methods

### Settings

Japan introduced the LTCI system in 2000. In the Japanese LTCI, all those aged 40 or older are asked to pay contributions (participation in the scheme is mandatory). Every individual aged 65 years or older and every individual aged 40 years or older with certain types of diseases are entitled to receive LTC services when assessed as needing LTC [2]. Under this system, a person’s available services are regulated by “care levels,” which is determined based on fair, objective, and nationally-standardized criteria (**Method A1**). Until 2006, these care levels comprised six LTCI categories: “support required level (SL),” “care required level (CL) 1,” “CL2,” “CL3,” “CL4,” and “CL5” in order of increasing severity (Individuals assigned to SL usually receive instrumental help, such as cleaning and shopping; in contrast, individuals assigned to CL5 are the most severe and receive support for basic activities of daily living, such as toileting and bathing). The scope of LTCI benefits is broad, and includes institutional care services and home-based care (e.g., home help, daycare, and temporary residential admission services). The coinsurance rate is 10% (except for few high-income recipients) until the upper limit determined by care levels, as recipients pay 100% of the fee for LTC services that exceed the upper limit (Additional file 1: Table S1).

LTCI became popular shortly after its introduction. However, an unexpectedly rapid increase in the demand for LTC was observed in the first several years; the number of beneficiaries increased from 2.2 million in 2000 to 4.1 million in 2005, and the cost of LTC service benefits soared from 3.6 trillion JPY (100 JPY = around 1 USD) in 2000 to 6.4 trillion JPY in 2005 [20]. Accordingly, the Japanese government made a major reform to the LTCI Law in 2006. This reform involved two major elements: First, for nursing home residents, it reduced economic incentives for institutionalization by imposing room and meal expenses (the out-of-pocket copayment rose by around 50% [21]). Second, for community-based care recipients as well, it reduced the availability of LTC services, aiming to prevent seniors at home with low care needs (i.e., SL or CL1) from becoming dependent (e.g., too much formal care would “spoil” care recipients and increase dependency) [21]. In the present study, we focus on the second element as an important policy change because we analyze caregivers who were providing care to community-based care recipients at home.

**Fig. 1:**
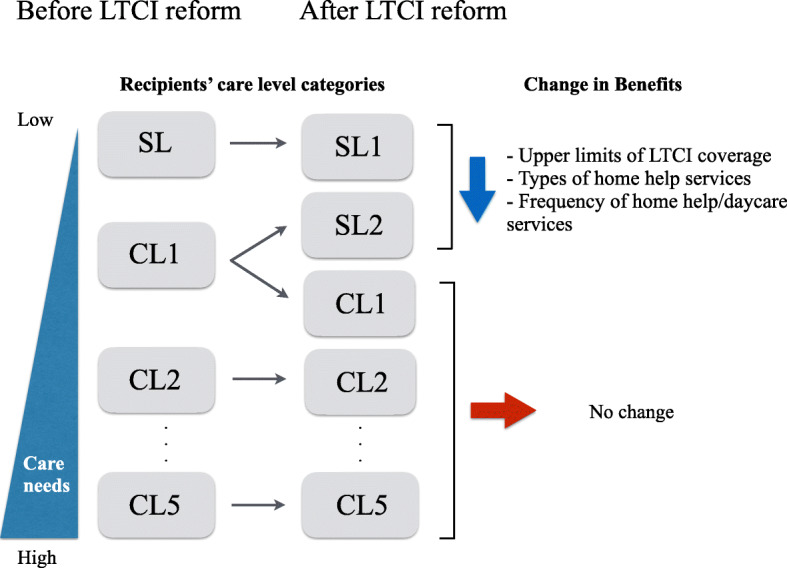
Schema of care level reclassification in the 2006 reform of the long-term care insurance in Japan. Notes: LTCI: long-term care insurance. Care levels consisted of “support required level (SL),” “care required level (CL) 1,” “CL2,” “CL3,” “CL4,” and “CL5” (in increasing order of severity) before the LTCI reform. After the reform, SL recipients were renamed SL1, and CL2–CL5 recipients stayed in the same category. CL1 recipients were re-categorized into a new category (SL2) if they were relatively healthy, but remained in CL1 otherwise

The details of the 2006 LTCI reform for community-based care recipients are explained below. It renamed “SL” to “SL1” without changing the criteria for categorization (Fig. 1). A new category “SL2” was added, which re-classified recipients who had previously been categorized as CL1 before 2006 to SL2 if they did not have dementia and their psychological or physical health status was not expected to worsen within the next 6 months (otherwise, the recipient remained in CL1). The reform reduced the volume and types of LTC services available to SL1 and SL2 recipients at home. First, the LTCI coverage upper limit was reduced by 19% for SL1 and 37% for SL2 (Additional file 1: Table S1). Second, fewer types of home help services were available than before 2006. For example, housekeeping and assistance with transport to/from hospital became unavailable for most SL1 recipients. Third, the frequency of available home help services and daycare was limited to twice per week for SL1 recipients and three times per week for SL2 recipients after 2006 (these were not limited before 2006). However, the reform did not change the types and volume of LTC services available for CL2–CL5 recipients at home. No other policy changes in the 2006 LTCI reform or later years affected either community-based SL/SL1 recipients at home or community-based CL2–CL5 recipients at home.

This policy change enabled us to apply a difference-in-differences (DID) approach by comparing informal caregivers of community-based SL/SL1 recipients (SL/SL1 caregivers) at home with informal caregivers of community-based CL2–CL5 recipients (CL2–CL5 caregivers) at home. In the main analysis, we excluded CL1 caregivers before 2006 and CL1 and SL2 caregivers after 2006 because the CL1 category before 2006 included recipients who would be assigned to SL2 after 2006 and those who would be assigned to CL1 after 2006, and we could not separate the former from the latter (In a sensitivity analysis, we conducted a robustness check using the sample including the CL1 caregivers before 2006 and CL1 and SL2 caregivers after 2006, and the results were qualitatively unchanged). It is known that a DID approach can be implemented using repeated cross-sectional data [22, 23], and this DID framework in the 2006 LTCI reform was used in a previous study [24].

### Data source

We used data from the Comprehensive Survey of Living Conditions (CSLC) [25], which is a nationally representative repeated cross-sectional survey of the non-institutionalized population in Japan, and has been used in previous studies [24, 26, 27]. The CSLC uses self-administered questionnaires to gather information on households and household members’ health every 3 years across all households selected by stratified cluster sampling (first stage included 600,000–800,000 people per year) and information on LTC for households selected from the first-stage sample by cluster sampling (including approximately 6000 people per year). The present study used data for households, household members’ health, and LTC in 2001 (wave 1), 2004 (wave 2), 2007 (wave 3), 2010 (wave 4), 2013 (wave 5), and 2016 (wave 6).

### Study sample

We first identified primary informal caregivers aged 30 years or older who provided care to co-resident family members aged 65 years or older. A primary informal caregiver was defined as a person who reported that he/she was making the largest contribution toward care or support for a dependent member in their household. Among these primary caregivers aged 30 years or older, we included caregivers of SL/SL1 recipients at home and caregivers of CL2–CL5 recipients at home by using LTC questionnaires (initial sample). Of the 14,256 caregivers in our initial sample, 1492 (10.5%) with at least one missing key variable were excluded. The remaining 12,764 caregivers comprised the analytic sample (individuals with missing variables: mean age 63.3 years and 72.0% women vs. analytic sample: mean age 64.3 years and 73.4% women). We defined SL/SL1 caregivers as the “treatment group” and CL2–CL5 caregivers as the “control group.”

### Outcomes: formal care use and intensity of informal caregiving

We examined four indicators of formal LTC service use, including the use of home help, daycare, and temporary residential admission services and the logarithm of out-of-pocket expenditure (thousand JPY) on formal LTC services. The indicators of the use of home help, daycare, and temporary residential admission were scored as 1 when the respondents answered the care recipients were using the service in May of the survey year. The out-of-pocket expenditure on formal LTC services included expenses on the services both within and beyond the LTCI coverage. We also examined an indicator of long-hours of informal caregiving (> 3 h per day) as the outcome indicating intensity of informal caregiving [28].

### Outcomes: caregiver health

Consistent with previous studies [9, 29, 30], we examined three health outcomes: 1) caregivers’ poor self-rated health status, 2) symptoms of a depressive state, and 3) symptoms of musculoskeletal diseases. Poor self-rated health was assessed with the question “How would you rate your current health status?” Possible answers were “Very good,” “Good,” “Moderate,” “Bad,” or “Very bad” [31]. Responses were dichotomized as 1 = “moderate,” “bad,” or “very bad,” and 0 = otherwise. Similarly, symptoms of a depressive state were scored as 1 when the respondents reported at least one instance of fatigue, insomnia, or appetite loss in the past few days, and 0 otherwise (These are part of the items that operationalized the diagnostic criteria for a major depressive episode outlined in Diagnostic and Statistical Manual of Mental Disorders [32]). A symptom of musculoskeletal disease was scored as 1 when the respondents reported at least one instance of stiff shoulders, back pain, or joint pain in the past few days, and 0 otherwise.

### Empirical strategies

#### Propensity score matching

A DID method can be applied to settings in which one group experiences a change in the treatment status (= the treatment group) while the other group does not (= the control group). A DID method assumes the time evolution of outcomes in the treatment group provides a valid counterfactual for the time evolution of outcomes in the treatment group absent the treatment (common trend assumption). To make the common trend assumption more credible, we applied a propensity score (PS) matching method [33, 34] to achieve covariate balance between the treatment group and the control group as well as between before and after the LTCI reform in 2006. To evaluate the PS of being assigned to the treatment group (vs. the control group), we used a logistic regression model that adjusted for factors related to either the probability of being treated or the outcome [35, 36], including indicator variables of waves, caregiver characteristics (gender, age, and marital status [married or not married]), and household characteristics (the number of household members [2, 3, 4, and 5+], an indicator of whether or not the household includes three generations, and the natural logarithm of equivalized household expenses [excluding LTC-related expenses] in Japanese yen [JPY]). Equivalized household expenses are calculated by dividing total expense in the same household by the root squared number of household members [37]. We supposed that household expenses better reflected SES than household income because more than half of primary informal caregivers were aged 60 or older and likely retired in Japan [25]. These characteristics of caregivers and households were included to construct the PS model because they are considered to affect informal caregiving status [27], as well as formal care use [38] and health outcomes [39, 40].

Based on the estimated propensity scores, we matched the treatment group and the control group using the kernel matching method with a bandwidth of 0.06 [33]. In kernel matching, weights are assigned to each individual in the control group based on their closeness to the nearest treatment. A value of 0 was assigned to off-support observations. The logistic regression models were refined by structured iterative approach to achieve the balance of covariates between the treatment group and the control group [41]. Standardized differences smaller than 0.10 are considered as negligible differences [35, 42].

#### Statistical analysis

We first described the characteristics of the analyzed caregivers and compared the treatment and control groups both before and after PS matching. Next, we depicted the trend of each outcome during 2001–2016 for the matched participants. We checked common trend assumptions before the LTCI reform in 2006 by visually comparing the outcome trends between the treatment and control groups.

Then, to estimate the effect of the LTCI reform on formal care use, intensity of informal caregiving, and the health outcomes, we used a DID approach for repeated cross-sectional data for the matched participants with the following equation [22, 43]:

$$ {Y}_i={\beta}_0+{\beta}_1\bullet {TREATMENT}_i+{\beta}_2\bullet T{REATMENT}_i\times 1{\left[ wave\ is\ after\ 2006\right]}_i+{\beta}_3\bullet 1{\left[ wave\ is\ after\ 2006\right]}_i+{\varepsilon}_i. $$

Here, subscript *i* indicates an individual. *Y*_*i*_ is individual *i* ’s outcomes. It should be noted that since our data are repeated cross-sectional data, an individual *i* is observed only once during the waves 1 to 6, and the subscript for time is not necessary (in contrast to a DID method for longitudinal data that follow up the same individuals). *TREATMENT*_*i*_ is a dummy variable that is scored 1 if the individual *i* is in the treatment group and 0 otherwise. 1[*wave is after* 2006]_*i*_ is an indicator that is scored 1 if the wave in which the individual *i* is observed is after 2006 (i.e., for waves 3–6) and 0 otherwise. *ε*_*i*_ is an idiosyncratic error term. We applied an ordinary least squares estimation with standard errors clustered by prefecture (*n* = 47) to account for a potential correlation of caregivers living in the same prefecture [43]. Under the common trend assumption stated above, the differences between the change over time for the treatment group vs. that for the control group represents the impact of the policies that change over time for the treatment group but not for the control group (i.e., the LTCI reform in 2006). In our regression model, the differences between the change over time for the treatment group vs. that for the control group are expressed by the coefficient of the interaction term between an indicator of the treatment group (*TREATMENT*_*i*_) and indicator of post-treatment (1[*wave is after* 2006]_*i*_)—i.e., *β*_2_. To make the presentation more accessible to readers, we showed *β*_2_ multiplied by 100 (except for the logarithm of out-of-pocket expenditure on LTC), which means by how many percentage points the LTCI reform relatively increased the percentage of experiencing each outcome in the treatment group compared to the control group (average treatment effect on the treated). Two-tailed *p*-values below 0.05 were interpreted as statistically significant in the main analyses.

### Secondary analyses

We conducted a series of post-hoc analyses. First, as a sensitivity analysis, we repeated analyses by using the expanded sample, including the sample in the main analysis plus the CL1 caregivers before 2006 and CL1 and SL2 caregivers after 2006. Second, we repeated analyses for subgroups stratified by age (≥65 years vs. < 65 years) and gender, because previous studies suggested the caregiving-related burden was concentrated in women or aged persons [44, 45]. In this analysis, the propensity scores were estimated for the subgroups stratified by age (≧65 years or < 65 years) and gender (women or men). For the gender-specific subgroups, gender was removed from the model to construct the propensity scores. Heterogeneity in the effect of the LTCI reform by subgroups was assessed by independent samples *t*-tests. All analyses were conducted using Stata 15 (College Station, TX; StataCorp LLC.).

## Results

### Caregivers’ characteristics

Of the 12,764 analytic caregivers, 2094 (16.4%) were in the treatment group, and 10,670 (83.6%) were in the control group. Before the PS matching, compared with the control group, the treatment group were slightly younger (mean age 63.0 years vs. 64.5 years), and a smaller proportion were women (68.2% vs. 74.5%) (Table 1). The treatment group were also more likely to have smaller number of household members. After the PS matching, three off-support caregivers (caregivers who did not match with any caregivers in the treatment group) were excluded from the analyses. In the PS matched sample, all the possible covariates were balanced well (standardized differences smaller than 0.10).

**Table 1:** Caregiver characteristics before and after propensity score matching

	Overall sample			Propensity score matched sample^a^		
	Treatment	Control	Standardized difference	Treatment	Control	Standardized difference
Number of caregivers	2094	10,670		2094	10,667	
Age, mean (SD), y	63.0 (12.7)	64.5 (11.6)	−0.12	63.0 (12.7)	63.5 (11.6)	−0.04
Women, %	68.2	74.5	−0.14	68.2	71.4	−0.07
Married, %	20.8	18.1	0.07	20.8	19.0	0.04
Number of household members, %						
2	37.6	32.6	0.11	37.6	36.1	0.03
3	25.2	26.9	−0.04	25.2	26.3	−0.03
4	14.9	16.5	−0.04	14.9	15.1	−0.004
5+	22.3	24.1	−0.04	22.3	22.6	−0.01
Monthly household expenditure, mean (SD), thousand JPY^b^	2.5	2.5	−0.06	2.5	2.5	−0.02
Three generation household, %	34.1	34.8	0.01	34.1	34.7	0.01

*SD* Standard deviation. The treatment group comprises SL/SL1 caregivers, and the control group includes CL2–CL5 caregivers

^a^3 off-support caregivers in the control group were excluded for the popensity score matched sample. The percentages for the control group in the matched sample were calculated according to weights assigned in the kernel matching

^b^Monthly household expenditure was equivalized by dividing total expense excluding out-of-pocket expenditure on long-term care services in the same household by the root squared number of household members

### Effect of the LTCI reform on formal care use and intensity of informal caregiving

**Fig. 2:**
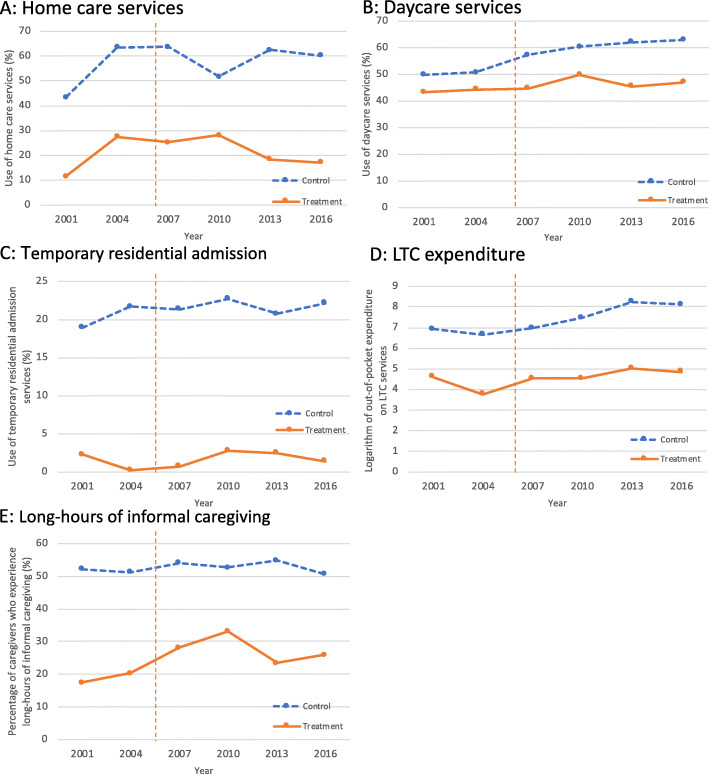
Trends of indicators of formal long-term care use and hours of informal caregiving from 2001 through 2016. Notes: (**a**, **b**, and **c**) show the trends of the percentage of caregivers who use home care, daycare, and temporary residential admission services, respectively, for the treatment group and the control group. The percentages were calculated for the propensity-score matched sample. **d** shows the trends of the logarithm of out-of-pocket expenditure on formal LTC services (thousand Japanese yen). **e** shows the trends of the percentage of caregivers who experienced long hours of caregiving (> 3 h per day). The vertical line indicates the long-term care insurance reform in 2006, which affected only the treatment group

For the PS matched sample, the trends of indicators of formal LTC use and long hours of informal caregiving from 2001 through 2016 appeared to be almost parallel before 2006, except for the use of temporary residential admission services (Fig. 2).

When we focused on the effect of the LTCI reform on the use of formal LTC services and intensity of informal LTC (Table 2) after 2006 for the PS matched sample, we found relative decreases in the use of home help services by 6.2 percentage points (95% confidence interval [CI]: 2.0–10.3; *p* < 0.01) and daycare services by 6.0 percentage points (95%CI: 1.1–10.9; *p* = 0.02), and a relative increase in the percentage of long hours of informal caregiving by 7.4 percentage points (95%CI: 0.2–14.5; *p* = 0.05) in the treatment group compared to the control group. The use of temporary residential admission services did not change significantly. We did not find any evidence of increased out-of-pocket expenditure on LTC services.

**Table 2:** Effect of the long-term care insurance reform on formal and informal care services use

Use of home help services (%)		Use of daycare services (%)		Use of temporary residential admission services (%)		Logarithm of LTC out-of-pocket expenditure		Long-hours of informal caregiving^a^ (%)	
DID^b^ (95% CI)	*P* value	DID^b^ (95% CI)	*P* value	DID^b^ (95% CI)	*P* value	DID^b^ (95% CI)	P value	DID^b^ (95% CI)	*P* value
−6.2 (−10.3, −2.0)	< 0.01	−6.0 (−10.9 to −1.1)	0.02	−0.5 (−2.5 to 1.5)	0.63	− 0.3 (− 0.7, 0.1)	0.18	7.4 (0.2 to 14.5)	0.05

*DID* Difference-in-differences, *LTC* Long-term care. ^a^Long-hours of informal caregiving indicate providing informal care more than 3 h per day. ^b^We analyzed the propensity score matched sample of 12,761 caregivers using an ordinary least squares regression with prefecture-level clustered standard errors. We showed the coefficient β_2_s multiplied by 100, except for logarithm of LTC out-of-pocket expenditure

### Effect of the LTCI reform on caregiver health

**Fig. 3:**
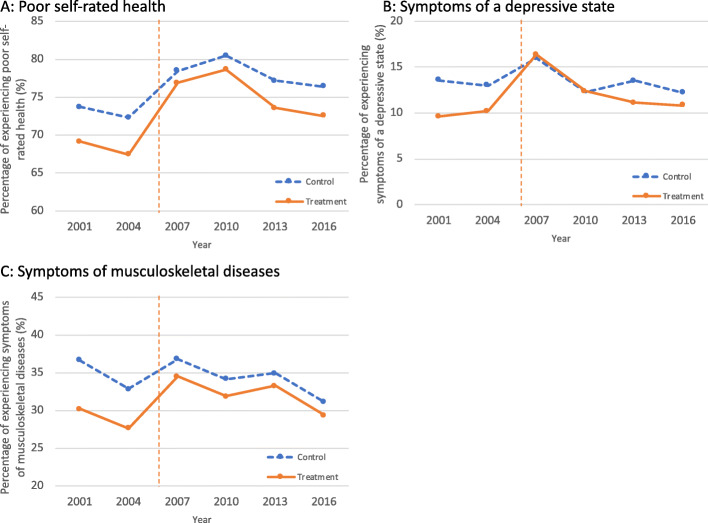
Trends of health outcomes among caregivers from 2001 to 2016. Notes: (**a**) shows the trends of the percentage of experiencing poor self-rated health for the treatment group and the control group. The percentages were calculated for the propensity-score matched sample. **b** shows trends experiencing symptoms of a depressive state, and (**c**) shows trends of experiencing symptoms of musculoskeletal diseases. The vertical line indicates the long-term care insurance reform in 2006, which only affected the treatment group

Figure 3 depicts the trends of the health outcomes from 2001 through 2016 for the treatment group and the matched control group. Although only two time points were observed before the reform, the outcome trends appeared to be almost parallel before 2006.

In DID analyses (Table 3), the effects of the LTCI reform on health for the treatment group compared to the control group were 2.2 percentage points higher on the percentage of those experiencing poor self-rated health (95%CI: 0.7–3.7; *p* = 0.01), 2.7 percentage points higher on the percentage of caregivers experiencing symptoms of a depressive state (95%CI: 0.5–4.8; *p* = 0.03), and 4.7 percentage points higher on the percentage of caregivers experiencing symptoms of musculoskeletal diseases (95%CI: 3.6–5.7; *p* < 0.001).

**Table 3:** Effect of the long-term care insurance reform in 2006 on health outcomes

Poor self-rated health (%)		Symptoms of a depressive state (%)		Symptoms of musculoskeletal diseases (%)	
DID^a^ (95% CI)	*P* value	DID^a^ (95% CI)	*P* value	DID^a^ (95% CI)	*P* value
2.2 (0.7 to 3.7)	0.01	2.7 (0.5 to 4.8)	0.03	4.7 (3.6, 5.7)	< 0.001

DID: difference-in-differences. ^a^We analyzed the propensity score matched sample of 12,761 caregivers using an ordinary least squares regression with prefecture-level clustered standard errors. We showed the coefficient β_2_s multiplied by 100, which showed by how many percentage points the long-term care insurance reform in 2006 increased the percentage of experiencing the outcomes (Null hypothesis: coefficient = 0)

### Secondary analyses

Our findings were qualitatively unchanged by the use of the expanded sample (Additional file 1: Table S2 and Table S3). In the stratified analyses, we found no evidence that the relative effects of the LTCI reform on caregivers’ formal LTC use and intensity of informal caregiving in the treatment group compared to the control group differed by gender and age group (Additional file 1: Table S4). As for the health outcomes, we found no evidence that the relative effects of the LTCI reform on caregivers’ health outcomes in the treatment group compared to the control group varied by gender (Additional file 1: Table S5). In contrast, when stratified by age group, we found the effect of the LTCI reform on self-rated health was more severe when caregivers were aged 65 years or older compared to when they were younger than 65 years (heterogeneity test, *p* < 0.001). The deteriorating effects on health conditions were especially found for caregivers aged 65 years or older (a 4.4 percentage points relative increase in experiencing poor self-rated heath for the treatment group compared to the control group [95%CI: 1.6 to 7.1; *p* < 0.01]).

## Discussion

In this quasi-experimental study using a large, representative sample of caregivers across Japan, we demonstrated that the 2006 LTCI reform relatively increased percentages of informal caregivers experiencing poor self-rated health, symptoms of a depressive state, and musculoskeletal disease among the caregivers for seniors with low care needs compared to caregivers for seniors with high care needs. The 2006 LTCI reform also had a decreasing effect on formal community-based LTC use and an increasing effect on the hours of informal caregiving. The out-of-pocket expenditure on formal LTC services did not increase. This indicated that when caregivers for seniors with low care needs became faced with reduced LTCI benefits, they decreased their use of formal LTC services and substituted informal LTC, rather than paying the full price for formal LTC services falling outside LTCI coverage. Taken together, these findings suggested that the reduced LTCI benefits for informal caregivers providing care to seniors with low care needs increased their caregiving burden, which might partly explain the deterioration of the multiple dimensions of health outcomes in this caregiver group.

There are several potential mechanisms through which reduced LTCI benefits in the LTCI reform deteriorated health status among community-based caregivers for seniors with low care needs. First, our results suggest that it may be explained partly by the increased hours of informal caregiving involved with reduced formal LTC use. The net effect of informal caregiving on health can be considered as a balance between the positive effects and the negative effects of informal caregiving [17]. On the one hand, according to the model of the impact of stress on health [46, 47], informal caregiving becomes hazardous to ones’ health when its psychological/physical demands exceed the “reserve capacity” of available psychological and social resources to cope [17]. On the other hand, altruism and satisfaction via providing help to others itself may be associated with better health outcomes including decreased depression and lower morbidity [14, 15]. Given this framework, increased hours of informal caregiving increased psychological/physical demands among informal caregivers, and as a result, the negative health effect of caregiving might have exceeded the positive health effect. Second, other dimensions of care-related burden in addition to caregiving hours might have increased; informal caregivers might have to perform more financial, physical, or emotional care-related support [48]. Moreover, faced with the limited social protection from public welfare programs, caregivers might feel more anxiety that they may not be able to use formal LTC services when they are necessary [49]. Third, reduced formal care availability might have adversely affected care recipients’ health status, which might subsequently have resulted in the deterioration of caregivers’ health [50, 51]. Another possible explanation is that the LTCI reform might affect the characteristics of the caregiver population (a selection effect). Among the potential caregivers for seniors with low care needs, those vulnerable to caregiving-related stress might be more likely to begin/continue to provide care after the LTCI reform compared to before the reform.

The adverse health effects of the LTCI reform were more severe among older caregivers especially in terms of self-rated health, while there was no evidence that the effect of the LTCI reform on formal LTC use and intensity of informal caregiving differed by the age groups. This may be because older caregivers might be more vulnerable to physical/psychological care-related burdens compared to younger caregivers because of limited physical functions and social support [52]. This finding suggests that access to formal LTC services should be guaranteed, especially among the elderly taking care of the elderly (described as the Japanese term *rou-rou kaigo* [53]). In contrast, we found no evidence that the effect of the LTCI reform on the intensity of informal caregiving or health outcomes varied by gender. Given some studies reporting that women are more likely to experience adverse health effects of intensive informal caregiving [9, 13], the effects of the LTCI reform on caregiver health might be explained not only by the change in the intensity of informal caregiving but also by other mechanisms.

Our study adds to the limited empirical studies investigating the benefits of formal LTC use on caregiver health. Some quasi-experimental studies conducted in European countries reported that the use of daycare did not significantly affect psychological and psychosomatic health among informal caregivers of older adults [54–56]. Regarding temporary residential admission, one UK study reported an increase in total sleep and subjective sleep quality per night during respite periods among caregivers of care recipients with dementia [57]. Another study showed that psychological distress increased following respite periods [58]. However, these previous studies were limited to small numbers of participants and only focused on specific types of formal care. Recently, Wagner and Brandt reported a cross-sectional positive association between regional-level LTC availability and spousal caregivers’ well-being across 11 European countries [30]. Our findings extend the findings of their study as we included participants from a different society.

Some limitations of this study should be noted. First, the present study is an observational study, and factors other than the reduced availability of community-based care might affect our findings. For example, the LTCI reforms in 2006 and later years restricted the availability of nursing home residence covered by the LTCI (as mentioned in the setting section), and an undersupply of nursing home beds compared to its demand is getting worse [59, 60]. These trends might have a selection effect on the informal caregiver population. Namely, it would force more caregivers who are providing care to seniors with high-level care needs (i.e., the control group) to continue care at home and therefore might worsen health status on average among the control group. However, if this is the case, this would bias our estimates towards the null, and the true effect of the reduced availability of LTC services on caregiver health would be larger than what we have estimated. Related to this, a DID approach for repeated cross-sectional data, as used in the present study, assumes that the pattern in the selection of the participants is similar over years. Even though we balanced caregiver characteristics between the treatment and the control groups across waves using PS matching, some degree of unobserved heterogeneity would be left inevitably. Thus, we could not completely distinguish the causal effect of the LTCI reform on caregivers from the selection effect on caregiver population. Second, the generalizability of our results to contexts outside Japan is unclear. Specifically, the caregiver burden in East Asian societies may be larger than in Western countries because Confucian cultures have a strong tradition of family responsibility for providing care that rests with middle-aged adult children [3, 61]. Therefore, the adverse effects of informal caregiving substituted for formal LTC may be more prominent when compared with Western countries. Third, the health outcomes evaluated in this study did not include objective health indicators. Finally, the long-term effects of the LTCI reform on caregivers’ health status should be explored in further studies.

## Conclusions

In summary, we found that reduced formal care availability following the Japanese LTCI reform increased hours of informal LTC corresponding to reduced use of formal LTC and deteriorated multiple dimensions of caregiver health. Our findings would highlight the importance of enhancing the availability of formal LTC services for both care recipients and caregivers and suggest the countries that are developing an LTCI system should not ignore the effect of formal LTC availability on caregiver health.

## Supplementary information

**Additional file 1: Method S1.** (Settings detail). **Table S1.** Upper limits in long-term care insurance (LTCI) coverage for recipients at home by care level before and after the LTCI reform in 2006. **Table S2.** Effect of the long-term care insurance reform in 2006 on formal and informal care services use: an analysis using the expanded sample. **Table S3.** Effect of the long-term care insurance reform in 2006 on health outcomes: total sample: an analysis using the expanded sample. **Table S4.** Effect of the long-term care insurance reform in 2006 on formal and informal care services using samples stratified by gender and age. **Table S5.** Effect of the long-term care insurance reform in 2006 on health outcomes: stratified by gender and age.

## Data Availability

The datasets generated and/or analyzed during the current study are not publicly available because the Comprehensive Survey of Living Conditions (CSLC) is conducted by the Japanese government, and permission is required to access the CSLC under the Statistics Act. For the current study, the Japanese Ministry of Health, Labour and Welfare approved the secondary use of the data (approval no. 1130–1). The aggregated data are available from the corresponding author on reasonable request.

## Abbreviations

CL: care required level
CI: confidence interval
CSLC: Comprehensive Survey of Living Conditions
DID: differences-in-differences
JPY: Japanese yen
LTC: long-term care
LTCI: long-term care insurance
PS: propensity score
SL: support required level
