# Supplementary material for: Effect of reduced formal care availability on formal/informal care patterns and caregiver health: a quasi-experimental study using the Japanese long-term care insurance reform
Source: BMC Geriatr. 2020 Jun 12;20:207. doi: 10.1186/s12877-020-01588-7 (PMC7291452; doi:10.1186/s12877-020-01588-7)
Supplement: Supplementary file 1 — Additional file 1: Method S1. (Settings detail). Table S1. Upper limits in long-term care insurance (LTCI) coverage for recipients at home by care level before and after the LTCI reform in 2006. Table S2. Effect of the long-term care insurance reform in 2006 on formal and informal care services use: an analysis using the expanded sample. Table S3. Effect of the long-term care insurance reform in 2006 on health outcomes: total sample: an analysis using the expanded sample. Table S4. Effect of the long-term care insurance reform in 2006 on formal and informal care services using samples stratified by gender and age. Table S5. Effect of the long-term care insurance reform in 2006 on health outcomes: stratified by gender and age. [file 12877_2020_1588_MOESM1_ESM.docx]

**Appendix**

**Effect of Reduced Formal Care Availability on Formal/Informal Care Patterns and Caregiver Health: A Quasi-Experimental Study Using the Japanese Long-Term Care Insurance Reform**

Method A1 (Settings detail).

Table A1. Upper limits in long-term care insurance (LTCI) coverage for recipients at home by care level before and after the LTCI reform in 2006

Table A2. Effect of the long-term care insurance reform in 2006 on formal and informal care services use: an analysis using the expanded sample

Table A3. Effect of the long-term care insurance reform in 2006 on health outcomes: total sample: an analysis using the expanded sample

Table A4. Effect of the long-term care insurance reform in 2006 on formal and informal care services using samples stratified by gender and age

Table A5. Effect of the long-term care insurance reform in 2006 on health outcomes: stratified by gender and age

**Method A1 (Settings detail).**

LTC services eligibility and care levels are determined as fairly and objectively as possible for every applicant according to a nationally standardized system. First, trained municipality staff visit homes and evaluate care needs using validated, structured instruments to determine the physical and mental status of applicants. Then, following the results, “estimated care minutes” are calculated by a computer-based needs assessment algorithm based on data collected for a large-scale study of professional caregivers in nursing homes. In other words, estimated care minutes can be regarded as a continuous indicator of care need level. After each applicant is automatically allocated to “non-eligible” or one of the “care levels” according to the estimated care minutes (initial assessment), the Care Needs Certification Board—a municipality committee consisting of health and other professionals—reviews the initial assessment considering the primary physician’s statement and changes the care level if necessary. Notably, the certification of care level does not hinge on the individual’s income, assets, or informal care availability.

**Table A1.** Upper limits in long-term care insurance (LTCI) coverage for recipients at home by care level before and after the LTCI reform in 2006

|  | Before LTCI reform | | After LTCI reform | |
| --- | --- | --- | --- | --- |
|  | Care level | Upper limits (JPY) | Care level | Upper limits (JPY) |
| (1) | SL | 61,500 | **SL1** | **49,700** |
| (2) | CL1 | 165,800 | **SL2** | **104,000** |
|  |  |  | CL1 | 165,800 |
| (3) | CL2 | 194,800 | CL2 | 194,800 |
| (4) | CL3 | 267,500 | CL3 | 267,500 |
| (5) | CL4 | 306,000 | CL4 | 306,000 |
| (6) | CL5 | 358,300 | CL5 | 358,300 |

LTCI: long-term care insurance. JPY: Japanese yen (100 JPY = around 1 US dollar). SL: Support required level. CL: Care required level. The upper limits increased by approximately 0.6% in 2014 in accordance with the increase in consumer tax. The care levels that experienced a reduction in the LTCI coverage are indicated in bold.

**Table A2.** Effect of the long-term care insurance reform in 2006 on formal and informal care services use: an analysis using the expanded sample

| Use of home help services  (%) | | Use of daycare services  (%) | | Use of temporary residential admission services  (%) | | Logarithm of LTC out-of-pocket expenditure | | Long-hours of informal caregiving^*^  (%) | |
| --- | --- | --- | --- | --- | --- | --- | --- | --- | --- |
| DID^†^  (95% CI) | P value | DID^†^  (95% CI) | P value | DID^†^  (95% CI) | P value | DID^†^  (95% CI) | P value | DID^†^  (95% CI) | P value |
| -0.3  (-3.4, 2.6) | 0.81 | -5.0  (-8.1 to -1.8) | <0.01 | -0.8  (-2.8 to 1.2) | 0.42 | -0.1  (-0.4, 0.2) | 0.39 | 5.9  (0.4 to 11.3) | 0.04 |

DID: difference-in-differences. LTC: long-term care. We analyzed 18,961 caregivers, including the sample in the main analysis plus the CL1 caregivers before 2006 and CL1 and SL2 caregivers after 2006. ^*^Long-hours of informal caregiving indicate providing informal care more than 3 hours per day. ^†^An ordinary least squares regression with prefecture-level clustered standard errors was conducted. We showed the coefficient β_2_s multiplied by 100, except for logarithm of LTC out-of-pocket expenditure.

**Table A3.** Effect of the long-term care insurance reform in 2006 on health outcomes: total sample: an analysis using the expanded sample

| Poor self-rated health  (%) | | Symptoms of a depressive state  (%) | | Symptoms of musculoskeletal diseases  (%) | |
| --- | --- | --- | --- | --- | --- |
| DID^*^  (95% CI) | P value | DID^*^  (95% CI) | P value | DID^*^  (95% CI) | P value |
| 1.9  (0.7 to 3.1) | 0.01 | -0.5  (-1.9 to 0.8) | 0.37 | 1.8  (-0.4, 4.1) | 0.08 |

DID: difference-in-differences. We analyzed 18,961 caregivers, including the sample in the main analysis plus the CL1 caregivers before 2006 and CL1 and SL2 caregivers after 2006. ^*^An ordinary least squares regression with prefecture-level clustered standard errors was conducted. We showed the coefficient β_2_s multiplied by 100, which showed by how many percentage points the long-term care insurance reform in 2006 increased the percentage of experiencing the outcomes (Null hypothesis: coefficient = 0).

**Table A4.** Effect of the long-term care insurance reform in 2006 on formal and informal care services using samples stratified by gender and age

|  |  | Use of home help services  (%) | | Use of daycare services  (%) | | Use of temporary residential admission services  (%) | | Logarithm of LTC out-of-pocket expenditure | | Long-hours of informal caregiving^*^  (%) | |
| --- | --- | --- | --- | --- | --- | --- | --- | --- | --- | --- | --- |
|  |  | DID^†^  (95% CI) | P value | DID^†^  (95% CI) | P value | DID^†^  (95% CI) | P value | DID^†^  (95% CI) | P value | DID^†^  (95% CI) | P value |
| By gender | Women  (n=9,375) | -5.0  (-9.7, -0.3) | 0.04 | -4.3  (-10.1 to 1.5) | 0.15 | -1.3  (-3.6 to 1.1) | 0.30 | -0.2  (-0.5, 0.5) | 0.95 | 6.1  (0.6 to 11.5) | 0.04 |
|  | Men  (n=3,386) | -11.5  (-20.3 to -2.8) | 0.01 | -9.4  (-18.6 to -0.3) | 0.04 | -0.4  (-4.0 to 3.1) | 0.21 | -1.0  (-1.8 to -0.2) | 0.01 | 7.4  (-4.2 to 18.9) | 0.16 |
|  | Heterogeneity test^†^ | P = 0.19 | | P = 0.35 | | P = 0.67 | | P = 0.06 | | P = 0.84 | |
| By age group | Age≧65  (n=6,183) | -6.9  (-13.7, -0.1) | 0.05 | -6.2  (-13.9 to 1.4) | 0.07 | -1.9  (-4.8, 1.0) | 0.20 | -0.5  (-1.1 to 0.2) | 0.16 | 4.1  (-10.6 to 18.8) | 0.51 |
|  | Age<65  (n=6,578) | -6.7  (-12.2 to -1.2) | 0.02 | -4.2  (-10.6 to 2.2) | 0.20 | 0.6  (-2.2 to 3.3) | 0.69 | -0.2  (-0.7 to 0.4) | 0.56 | 9.5  (3.8 to 15.3) | <0.01 |
|  | Heterogeneity test^†^ | P = 0.96 | | P = 0.69 | | P = 0.21 | | P = 0.44 | | P = 0.49 | |

DID: difference-in-differences. ^*^Long hours of informal caregiving indicate providing informal care more than 3 hours per day. ^†^An ordinary least squares regression with prefecture-level clustered standard errors was conducted. We showed the coefficient β_2_s multiplied by 100, except for logarithm of long-term care out-of-pocket expenditure (Null hypothesis: coefficient = 0).

**Table A5.** Effect of the long-term care insurance reform in 2006 on health outcomes: stratified by gender and age

|  |  | Poor self-rated health  (%) | | Symptoms of a depressive state  (%) | | Symptoms of musculoskeletal diseases  (%) | |
| --- | --- | --- | --- | --- | --- | --- | --- |
|  |  | DID^*^  (95% CI) | P value | DID^*^  (95% CI) | P value | DID^*^  (95% CI) | P value |
| By gender | Women  (n=9,375) | 2.0  (0.7 to 3.2) | 0.01 | 2.4  (-0.4 to 5.2) | 0.08 | 5.0  (2.7 to 7.3) | <0.01 |
|  | Men  (n=3,386) | 2.3  (0.4 to 4.2) | 0.03 | 2.6  (-0.5 to 5.6) | 0.08 | 2.3  (-0.7 to 5.3) | 0.11 |
|  | Heterogeneity test^†^ | P = 0.79 | | P = 0.92 | | P = 0.15 | |
| By age group | Age≧65  (n=6,183) | 4.4  (1.6 to 7.1) | <0.01 | 5.1  (-0.6 to 10.8) | 0.07 | 4.5  (-0.5 to 9.5) | 0.07 |
|  | Age<65  (n=6,578) | -1.2  (-2.9 to 0.5) | 0.12 | 0.5  (-3.1 to 4.1) | 0.73 | 3.2  (-1.4 to 7.8) | 0.14 |
|  | Heterogeneity test^†^ | P < 0.001 | | P = 0.17 | | P = 0.70 | |

DID: difference-in-differences. ^*^An ordinary least squares regression with prefecture-level clustered standard errors was conducted. We showed the coefficient β_2_s multiplied by 100, which showed by how many percentage points the long-term care insurance reform in 2006 increased the percentage of experiencing the outcomes (Null hypothesis: coefficient = 0). ^†^For the stratified analyses, we showed p-values for the heterogeneity test assessed by independent samples *t*-tests for each category and each outcome (e.g., the p-value for the heterogeneity test in the effects on poor self-rated health for women vs. men was 0.79).
